# 4SpecID: Reference DNA Libraries Auditing and Annotation System for Forensic Applications

**DOI:** 10.3390/genes12010061

**Published:** 2021-01-02

**Authors:** Luís Neto, Nádia Pinto, Alberto Proença, António Amorim, Eduardo Conde-Sousa

**Affiliations:** 1 Campus de Gualtar, Rua da Universidade, 4710-057 Braga, Portugal; lmpneto137@gmail.com (L.N.); aproenca@di.uminho.pt (A.P.); 2 Instituto de Investigação e Inovação em Saúde (i3S), Universidade do Porto, Rua Alfredo Allen 208, 4200-135 Porto, Portugal; npinto@ipatimup.pt (N.P.); econdesousa@gmail.com (E.C.-S.); 3 Institute of Pathology and Molecular Immunology from University of Porto (IPATIMUP), Rua Júlio Amaral de Carvalho 45, 4200-135 Porto, Portugal; 4 Center of Mathematics of the University of Porto, Faculty of Sciences of University of Porto, Rua do Campo Alegre, 4169-007 Porto, Portugal; 5 Department of Informatics, Campus de Gualtar, University of Minho, Rua da Universidade, 4710-057 Braga, Portugal; 6 Faculty of Sciences of University of Porto, Rua do Campo Alegre, 4169-007 Porto, Portugal; 7 Instituto de Engenharia Biomedica (INEB), Universidade do Porto, Rua Alfredo Allen 208, 4200-135 Porto, Portugal

**Keywords:** barcoding, DNA, species identification, forensics, database auditing, efficient software, taxonomy, genetics, BOLD system

## Abstract

Forensic genetics is a fast-growing field that frequently requires DNA-based taxonomy, namely, when evidence are parts of specimens, often highly processed in food, potions, or ointments. Reference DNA-sequences libraries, such as BOLD or GenBank, are imperative tools for taxonomic assignment, particularly when morphology is inadequate for classification. The auditing and curation of these datasets require reliable mechanisms, preferably with automated data preprocessing. Software tools were developed to grade these datasets considering as primary criterion the number of records, which is not compliant with forensic standards, where the priority is validation from independent sources. Moreover, 4SpecID is an efficient and freely available software tool developed to audit and annotate reference libraries, specifically designed for forensic applications. Its intuitive user-friendly interface virtually accesses any database and includes specific data mining functions tuned for the widespread BOLD repositories. The built tool was evaluated in laptop MacBook and a dual-Xeon server with a large BOLD dataset (*Culicidae,* 36,115 records), and the best execution time to grade the dataset on the laptop was 0.28 s. Datasets of *Bovidae* and *Felidae* families were used to evaluate the quality of the tool and the relevance of independent sources validation.

## 1. Introduction

Over the years, DNA-based taxonomy has been gaining importance in a wide range of biological problems, as in species protection, monitoring and conservation, and detection of cryptic and nonindigenous species. In the forensic field, species (or higher level taxa) identification is a key issue in increasingly common problems involving food-fraud, wildlife protection, biocrimes, bioterrorism, or trafficking of species, either specimens or products [1]. Indeed, in many litigations, the identification of the species cannot be assessed by morphologic analysis, as the available evidence is not a whole organism, but (often highly processed) parts of it: pelts, skins, meat, teeth (ivory), horns (sometimes already powdered when seized). This is the most common situation in food fraud investigations, but other situations may arise, such as in illegal fishing and hunting, or when potions, ointments, or other “medicines” are investigated under the context of crimes against wildlife.

Short DNA sequences, known as barcodes, have been proved as powerful identification tools, due to high interspecies but low intraspecies variability, and have been widely used to species’ identification, definition, and delimitation [2]. Reference DNA libraries, such as GenBank [3,4] or BOLD [2], are indispensable tools for taxa identification, allowing the collection and organization of a huge amount of DNA sequences (over 8.9 M at BOLD) and associated metadata.

The success of the studies where DNA-based taxonomy is the only operative tool mainly depends on the accuracy and representativeness of the reference libraries of DNA sequences, which result from the contribution of the general scientific community. The drawback of this collaborative effort is that public databases are susceptible to inaccuracies or errors, such as poor quality of the molecular (meta)data or imprecise or even conflicting taxonomic identifications. To identify misleading data records, regular auditing of DNA sequences and corresponding annotation is required [5,6]. Despite already existing curated databases for specific taxa, as is the case of the UNITE database [7] or the several R-Syst databases (R-Syst::diatom, R-Syst::Bacteria, R-Syst::Virus, R-Syst::Plant, R-Syst::Fungi, R-Syst::Nematodes or R-Syst::Arthropods) [8,9], efficient and general algorithms for auditing and annotation of public databases are still needed (see [5,6], e.g.). Recently, automated and generalized algorithms were developed considering a ranking system primarily prioritized by the number of uploaded sequences (BAGS [10] and a MATLAB toolbox [11]), a criterion that is not in compliance with forensic golden standards, namely, when the set of sequences is deposited by a single source. The implementation of forensically oriented protocols and auditing algorithms is still lacking and their urgent development is imperative, privileging the quality, reproducibility and consistency of independently produced data.

This work presents 4SpecID, an efficient forensics-oriented tool that semiautomates the auditing and annotation of (remote or local) DNA libraries to ensure the quality standards of the compiled data for forensic applications, particularly assuring congruent data resulting from independent sources. It supports human interaction to refine the auditing and annotation processing with a very fast grading of records.

Specifically, 4SpecID is a user-friendly software tool, freely available at https://4specid.github.io, with source code, installers, and tutorials. It can deal with any library and works under a graphical environment based on graph theory, which allows the easy search, detection, and visualization of incongruent data. To illustrate 4SpecID performance we analyzed in this work the data of the *Bovidae* and *Felidae* families, retrieved from BOLD on November 2020, as well as the *Culicidae* family, retrieved from BOLD on March 2020. The results demonstrate the need and relevance of 4SpecID to help and orient experts in the auditing and annotating of DNA databases for taxonomic identification, and the extreme efficiency of the software tool during the auditing and annotation operations of very large databases. Moreover, 4SpecID prioritizes the database evaluation by the existence of independent sources of congruent data, according to forensics standards.

## 2. Material and Methods

### 2.1. The Tool 4SpecID 

Specifically, 4SpecID is a computational tool to audit and annotate reference DNA libraries. It automatically accesses, selects, aggregates, updates, and evaluates database records to validate the congruence between taxonomic data and genetic clustering. Furthermore, 4SpecID was specifically tailored for forensic applications, privileging above any other criterion the upload of data into the DNA libraries by independent sources. This tool guides the user to (visually) identify erroneous or ambiguous data records, which can be either removed or fixed, resulting, from this action, a curated database for a specific taxon. Although 4SpecID works for any specific taxon, we hereafter refer to species level for simplicity sake.

The current development of an efficient and portable software tool requires an adequate selection of the programming environment to best use the available hardware equipment. The starting material for this tool was a MATLAB script to audit and annotate records from a DNA library but without support for interactive and graphical editing of the available data [11]. This script was very slow to perform its tasks and with limited capabilities. The initial efforts started with a port with key modifications of this MATLAB auditing algorithm into a C++ program built with associated highly efficient libraries, consequent validation, and followed by performance improvements of the resulting C++ code of 4SpecID.

**Figure 1:**
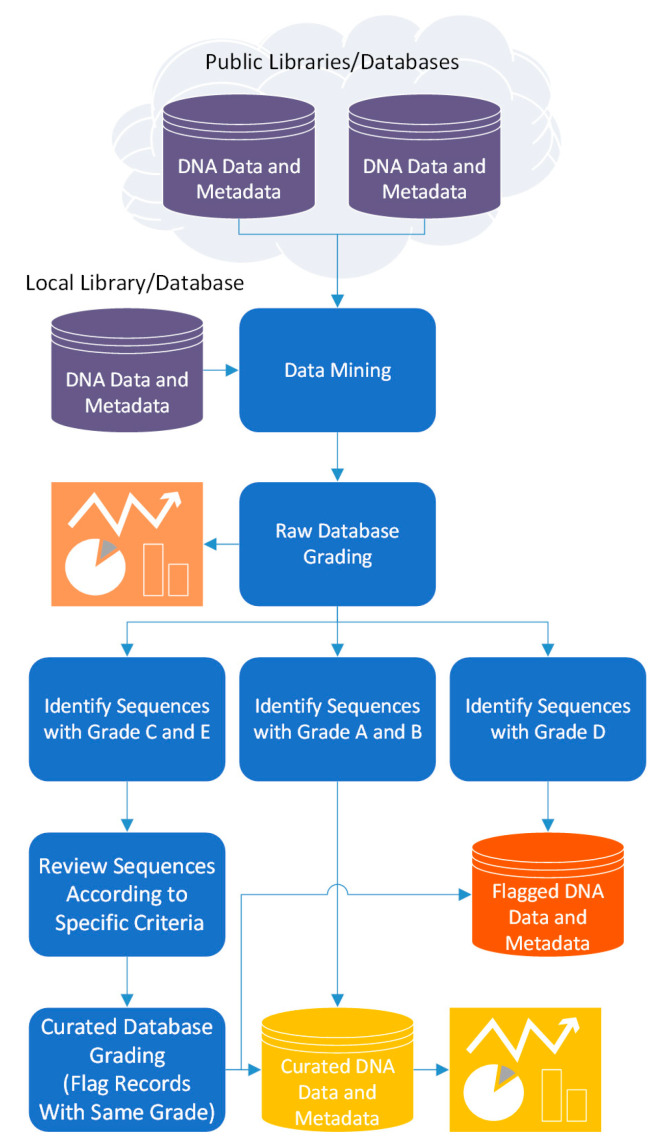
The pipeline flow of the 4SpecID computational tool.

Thus, 4SpecID pipeline to audit and annotate DNA libraries considers five steps (Figure 1):

(i) data mining: build the reference library dataset to audit, and the distance matrix from the information on clustering distance divergence
(ii) raw database grading: assignment of grades to input database records
(iii) semiautomated data curation: fixing and/or removal of database records considered unreliable by the auditor
(iv) curated database grading: assignment of grades to all records in the database resulting from previous step; and
(v) output generation: export the curated database and associated tables and statistics.

#### 2.1.1. Grading Algorithm: Principles

Briefly, 4SpecID grading algorithm considers five levels of accuracy and is primarily based on the principle that the same set of outcome results is more reliable if results have multiple independent sources than if they have the same single source. Rooted in this principle, 4SpecID evaluates the database records at two different levels: (i) if the records of a specific species (or other taxon) have more than a specified number of independent sources and (ii) if the taxonomic labeling of the records are in concordance with the genetic clustering.

The first level of evaluation addresses the number of independent sources owning the physical vouchers corresponding to a specific species. The records with less than n (user-definable parameter, default value = 2) independent voucher owners will be graded as D: insufficient independent sources. Indeed, regardless of the number of specimens associated with a specific species, a set of records from one single source lacks independent validation, being particularly vulnerable to systematic errors of the vouchers’ owner. D graded records should then not be used or at least considered with caution, under special circumstances.

The second level of evaluation relates to the concordance between species-level identification of the records and DNA clustering outcomes. DNA clustering algorithms have been used to group DNA samples into operational taxonomy units (OTUs) that closely match species-level taxonomy groups [12]. When the complete set of specimens of a given species on a dataset has a biunivocal relation with one single genetic clustering group, the reliability of the correspondent taxonomic classification can be considered higher than otherwise.

Based on these assumptions, 4SpecID classifies each taxon in the database into one out of five grades, from A, the most reliable, representing species from multiple independent sources where a biunivocal relationship between taxonomic identification and clustering group exists, to E, when incongruences between taxonomic identification and clustering groups are detected.

#### 2.1.2. Grading Algorithm: Dataset Representation

**Figure 2:**
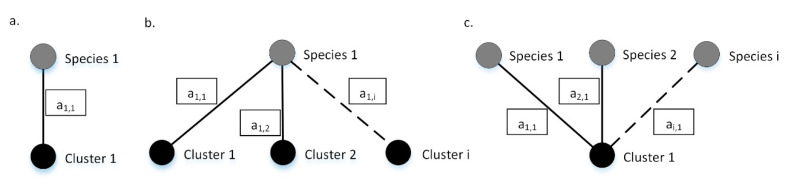
Example graphs. Species (in gray) and the corresponding clustering groups (in black) are connected through weighted edges. (**a**) All the a1,1 records identified as specimens of Species 1, are grouped on Cluster 1 and vice versa; (**b**) Species 1 contains a1,1, a1,2, and a1,i specimens grouped on clusters 1, 2,…, i, respectively; (**c**) Cluster 1 contains a1,1, a2,1, and ai,1 specimens of species 1, 2, and i, respectively. Dashed lines represent connections that may exist but are not mandatory to describe the example. The values inside each box represents edges weights.

The grading algorithm behind 4SpecID uses weighted undirected graphs where nodes represent species and clustering groups, and the weighted edges represent records in the database. Any edge must connect one species label to one clustering group. Edges are weighted, representing each weight the number of records in the dataset sharing species label and clustering group. As so, an edge will connect the nodes “Species *i*” and “Cluster *j*” with weight “$a_{i,j}$” if and only if there are $a_{i,j}$ database specimens labeled as “Species *i*” and grouped into “Cluster *j*” (see Figure 2).

Any dataset can be represented by a graph built as described, which will be comprised by sets of isolated subgraphs or components that are equivalent to one of the three described in Figure 2, or a combination of those. Ideally, there is a biunivocal relation between one species and one clustering group, and the isolated subgraph representing that specific species will have just two nodes: the species and the corresponding clustering group (Figure 2a). If specimens of one species are represented in more than one cluster, then the isolated subgraph representing that specific species will have more than two nodes: at least the starting species and two or more clustering groups (Figure 2b). If more than one species is clustered into one single clustering group, then the isolated subgraph will have three or more nodes: at least one clustering group and two species (Figure 2c).

#### 2.1.3. Grading Algorithm: Workflow

**Figure 3:**
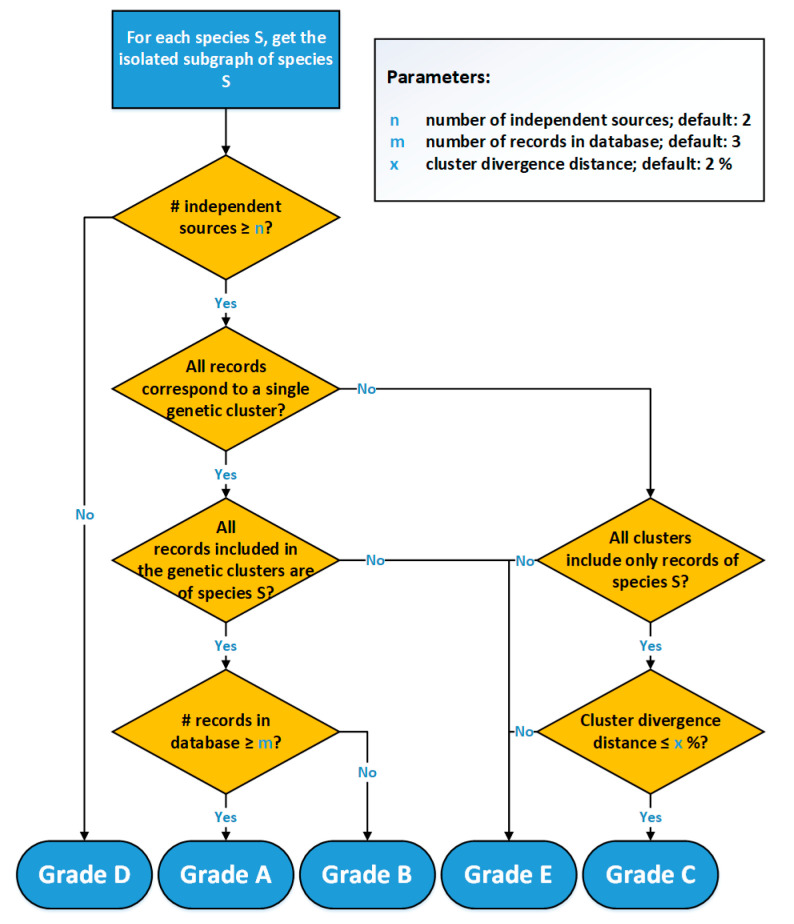
Flowchart of the 4SpecID implementation of the automated grading algorithm.

The grading workflow of 4SpecID (Figure 3) starts by assessing, for each species, the number of independent sources that own the physical vouchers, grading as D: “Insufficient Independent Sources,” those with less than n independent sources (a user-defined parameter, default = 2). In the second step, the graph representing all the entries in the dataset is built, and isolated subgraphs are considered one at the time.

All species with a sufficient number of sources represented by subgraphs equivalent to Figure 2a will be graded as A or B, depending on the weight of the edge: A in case of the number of records being equal or greater than *m,* B otherwise. All species represented by subgraphs equivalent to Figure 2b (one single species and connected to more than one cluster) will be graded as C or E: C if the cluster divergence distance is smaller than x (user-defined parameter x, default = 2%), E otherwise. All species represented by subgraphs equivalent to Figure 2c (one single cluster and connected to more than one species) will be graded as E.

#### 2.1.4. Input Files

The grading algorithm requires two types of input data: a species dataset and the divergence distances between targeted genetic clusters. Specifically, 4SpecID expects these input data in two text files (semicolon or tab-separated files), which should be, in principle, supplied by the user.

The first file is the dataset under evaluation, with as many fields as specified by the user, but where three are mandatory: (i) the species information, (ii) the genetic cluster identification, and (iii) the identification of the physical voucher owner.

The second file complements the dataset with information on the divergence distances between the targeted clusters. This is required when one single species is connected to more than one genetic cluster (as shown in Figure 2b). Briefly, 4SpecID was built to deal with any DNA reference library, either public, such as BOLD [2] or GenBank [3,4], or private ones. Since we anticipate that many users will use the BOLD database, 4SpecID uses a data mining module to automatically download clustering distances from BOLD, when necessary. In this case, the user does not need to supply this second input file, since the data mining module can do it; however, an internet connection is needed to download the data.

#### 2.1.5. Graphical User Interface

Genetic domain-experts often need to modify or correct in run-time the existing records when auditing them. This editing operation is simplified with a visual graphical interface, namely, when it supports textual modifications of data in records or directly editing of a visual graph with the species, genetic clusters, and associated edges connecting them. Moreover, 4SpecID was designed to be used by any researcher with no bioinformatics expertise, in contrast with other packages (e.g., [10,11]). Its flexible graphical user interface (GUI) offers the user access to several features without using command lines: it easily identifies and edits erroneous records and visualizes dataset records. It also generates and displays predefined statistics.

**Figure 4:**
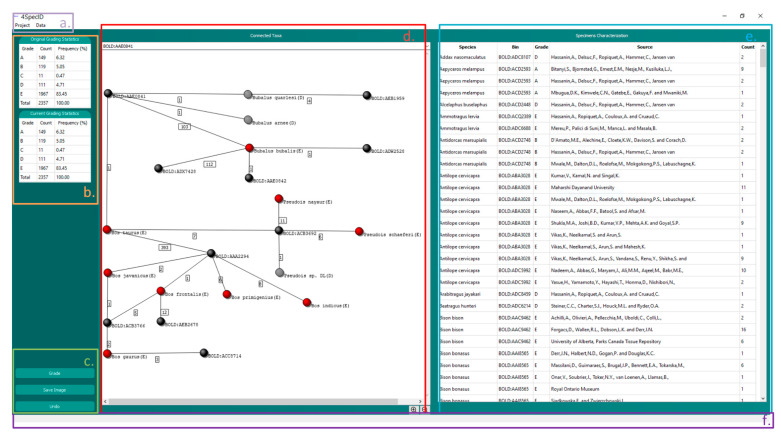
The 4SpecID User Interface: *(***a***)* Navigation Bar; *(***b***)* Statistical Results; *(***c***)* Action Buttons; *(***d***)* Graph Visualizer: nodes representing genetic clusters are colored in black, and nodes representing species graded as D and E are colored in gray and red, respectively; *(***e***)* Record Editor; and *(***f***)* Status Viewer.

The GUI in 4SpecID displays six blocks of data (see below and in Figure 4):

- NAVIGATION BAR: contains all actions to create, load, save, and delete projects, as well as to export results. This component also handles the action of loading distance matrices and conditional data filtering and defines auditing parameters.
- STATISTICAL RESULTS: displays the number and percentage of species within each grade.
- ACTION BUTTONS: buttons to grade all records, save graph from GRAPH VISUALIZER, and undo previous steps.
- GRAPH VISUALIZER: represents a subsample of the dataset as a graph. The user can change the subset by selection of a record on the RECORD EDITOR or through the search bar. This component can also be used to delete records identified by the user as unreliable. Nodes representing genetic clusters are colored in black, and nodes representing species graded as A, B, C, D, and E are colored in green, blue, yellow, gray, and red, respectively. The grade attributed to the species is also represented inside parentheses after its name.
- RECORD EDITOR: represents the dataset in the form of a spreadsheet and allows the user to modify, delete, or sort records.
- STATUS VIEWER: shows a textual feedback to the user when the application is performing a task.

### 2.2. Case Studies: Bovidae and Felidae families

To test and validate the 4SpecID tool, two datasets were selected: *Bovidae* and *Felidae* families, respectively, downloaded from BOLD Systems on 10 and 17 November 2020, both available at https://4specid.github.io. The choice of these families was based on the following criteria:

(a) Diversity of member species
(b) Degree of research, namely, taxonomic coverage
(c) Inclusion of wild and domesticated taxa
(d) Forensic interest (members involved in protection and *Bovidae* in food authenticity).

Both databases were downloaded from BOLD Systems public API by searching for all records with keywords “Bovidae” and “Felidae.” To ensure that none of the represented BINs (BOLD genetic clusters) [12] contained records from species not belonging to these families, a loop over all BINs was performed by downloading all records with each BIN as keyword. The resulting files were then concatenated and the duplications removed. Since BOLD Systems mine records from GenBank, the information regarding the owner of those records was not available in the downloaded file. To access these data, the Entrez Programming Utilities (E-Utilities) was used and the databases were updated with the physical voucher owner names. Finally, all records that were not from mitochondrial cytochrome c oxidase subunit I (COI) were removed from databases. The described data mining process was fully automated and the used Linux scripts are available at the 4SpecID webpage.

After the creation of a new project and the uploading of the dataset, 4SpecID automatically filters all records with insufficient required data. Records with missing information, such as species name, genetic cluster identification, or identification of the source, are automatically removed. After this first filtering, *Bovidae* dataset ended with 2357 records (from the original 2502) and *Felidae* with 565 (from the original 596).

## 3. Results

### 3.1. Performance Evaluation

Briefly, 4SpecID, a cross-platform software tool, was developed from an initial algorithm implementation in MATLAB (with no graphics interface). The development aimed performance portability with an integrated user-friendly graphics interface, by exploring enhanced parallel features. Two different Unix computing platforms were used to evaluate performance: (i) a simple laptop (a 2015 MacBook (Apple, Cupertino, CA, USA)) with a 2-core Intel Broadwell CPU with 8 GiB RAM and (ii) a dual-socket server in a computer cluster, with 2012 12-core Xeon Ivy Bridge devices and with 64 GiB RAM. Both Intel devices support 2-way simultaneous multithreading (SMT): up to 4 threads can be simultaneously executed on this MacBook, and up to 48 threads can simultaneously be active on this Xeon server. The code was compiled with Clang 10.0.1 (in the MacBook) and GCC 7.2.0 (in the server), both with all relevant optimization switches.

Execution times were the basic metric that was measured and each presented result is the average of the 5 best execution times within 5% difference error, in 8 runs. The measured execution times included the remote access to the BOLD web platform to retrieve the clustering distances, through a shared network service (with nondeterministic time responses). These accesses are only required when the tool is executed for the first time for a given BIN; the next time the user needs to access the same BIN, after editing a record, for instance, the tool automatically recovers the saved distance matrix, with a massive speedup of the auditing operation. To distinguish these two different runs of the tool (with different execution times), we designated the first as a “cold run” and the second as “warm run.”

A larger specimen family was selected for the quantitative evaluation of the tool: the *Culicidae* dataset, downloaded from BOLD in March 2020, with 36,115 records, representing 5220 species grouped into 5287 genetic clusters.

Several comparative performance evaluations are presented next:

- sequential evaluation: to show the impact of the selection of an adequate programming environment and the introduced code and data structure modifications to improve sequential code execution time
- parallel code scalability: to show how the increase in the number of simultaneous threads execution (using the available computing cores in the system) impacts the overall performance
- Finally, 4SpecID vs. competition: to show how the execution times of the new tool behave in comparison with the direct competition.

#### 3.1.1. Sequential Evaluation

The first C++ version was a straight port of a slightly modified MATLAB version, where key variables were restructured to improve the data traversal. This version received later some improvements: more efficient data structures to reduce the complexity of search operations and use of the well-tuned graph functions from the Boost library.

To evaluate the performance of these sequential versions, their execution times were measured on the laptop MacBook, including remote accesses and the reading of input files. The original MATLAB version ran in 70 min, while the original C++ version took 40 min and the improved version went down to 38 min.

#### 3.1.2. Parallel Code Scalability

Both target platforms are based on 2-way SMT multicore devices sharing a common memory. The development of code for these platforms must take advantage of parallel code in a shared memory environment, namely, with multithreaded parallel programs. Additionally, 4SpecID tool implementation followed this approach and used the thread pool class from the Boost library for thread management.

**Figure 5:**
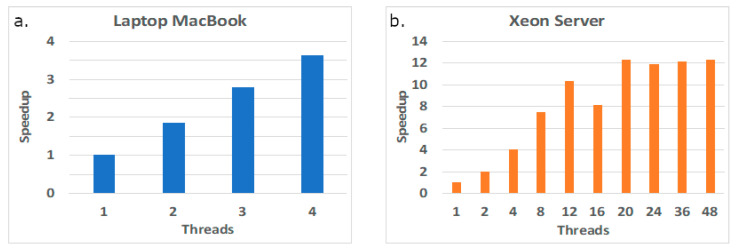
Performance scalability in two computing platforms: laptop MacBook (**a**) and Xeon server (**b**).

The set of measured execution times displayed in Figure 5 below aims to show if the performance of the tool in both platforms (MacBook and Xeon server) can be improved simply by increasing the number of cores. The plots taken in the laptop platform in Figure 5 show that the tool scales well with the number of threads, even when using the multithreaded feature at each core (up to two simultaneous threads per core). However, in the server platform, the tool scales well while in the first Xeon device (first 12 threads), but it then slowly improves the execution times when using the cores in the second Xeon device, suggesting a penalty due to data accesses, mostly due to the NUMA architecture of the node and to remote web accesses.

#### 3.1.3. 4SpecID vs. Competition

The main competitor of 4SpecID is BAGS [10], although we may also consider the original version in MATLAB [11] as another alternative worth analyzing its performance. Both competitors have limited capabilities when compared to our tool, as seen before, and the same applies to performance figures. Next comparative measurements were taken on the laptop.

The measured execution times of 4SpecID included two different situations: the cold run when the tool is executed for the first time for a given dataset—which require several remote accesses to get the required information to build the distance matrices between clusters—and the warm run when the distance matrices are already built and locally saved. This latter case only requires remote access to the remote database when the time stamp show it is overdue, and as a consequence, the warm run is considerably faster.

**Figure 6:**
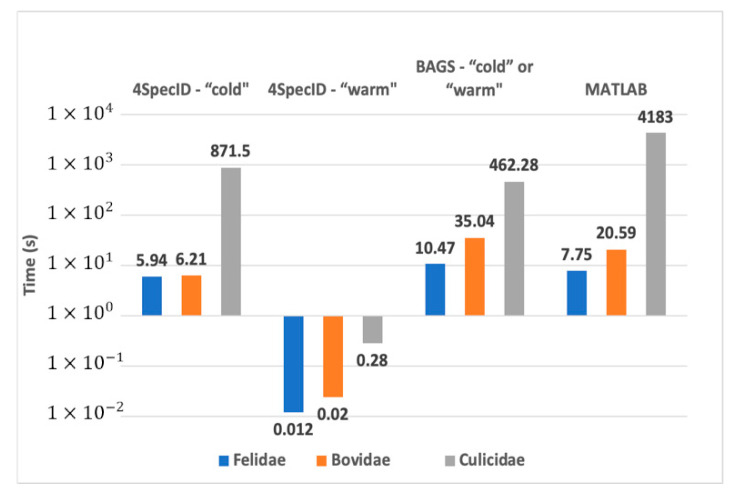
Comparative performance on the laptop: best execution times for a cold and a warm 4SpecID runs compared to BAGS and the original MATLAB version.

Measured results are shown in Figure 6. For the larger family, the *Culicidae* dataset, for each editing operation, the original MATLAB version needed 70 min, BAGS required 7.7 min, and 4SpecID performed that operation in 0.28 s!

### 3.2. Case Studies

For each project, 4SpecID requires 3 user-definable parameters: n, the minimum number of independent voucher owners or sources; m, the minimum number of records of a given species; and x, the maximum divergence distance between two clusters. For the two case studies presented next, the following parameters were assumed: n=2, m=10, and x=2%.

#### 3.2.1. Bovidae Family

**Figure 7:**
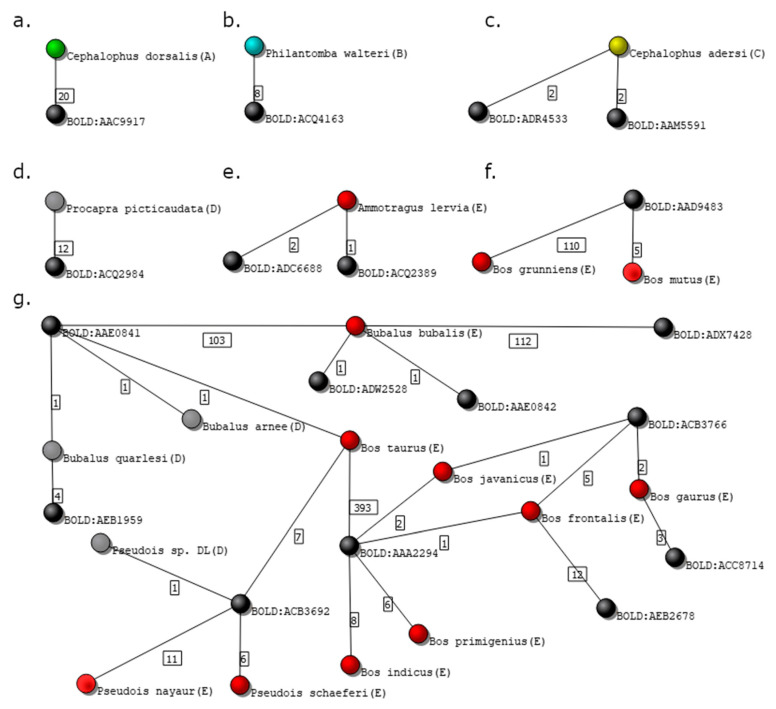
Isolated subgraphs from the Bovidae family dataset. Black nodes represent genetic clusters, while nodes colored in green, blue, yellow, gray, and red represent species graded as A, B, C, D, and E, respectively. (**a**) Species graded with A; (**b**) species graded with B; (**c**) species graded with C; (**d**) species graded with D; (**e**,**f**) species graded with E; and (**g**) complex isolated subgraph representing several species graded with D and E.

The 2357 *Bovidae* records represented 141 species, grouped into 151 BINs, and containing a large majority of unreliable records, most of them graded in the lowest category, E (see Table 1). Figure 7 presents examples of species assigned to the five possible grades.

Figure 7a shows the isolated subgraph of *Cephalophus dorsalis*, graded with A. The number of independent voucher owners of the 20 specimens presented in the database of *C. dorsalis* is 5, and the isolated subgraph contains only two nodes, the species, and the correspondent cluster, BOLD:AAC9917.

The species *Philantomba walteri* in the isolated subgraph in Figure 7b was graded as B, despite having just two nodes, since the number of analyzed records (8) is smaller than the value previously defined (*m* = 10).

Records of species *Cephalophus adersi* in the isolated subgraph in Figure 7c are grouped in two different clusters: BOLD:ADR4533 and BOLD:AAM5591. This is a particularly interesting example, as two clusters associated with a single species may had led to a grade E if the genetic distance between the clusters was greater than the one previously defined by the user (x=2%, in this case). Indeed, a grade C was assigned to the species because 4SpecID searched for the divergence distance between the clusters at the BOLD website and found that the distance between the BINs at stake is 1.44%, and thus smaller than x.

Figure 7d shows the two-node graph of species *Procapra picticaudata*, visually equivalent to those in panels a and b. However, since all 12 records were obtained from one single source, the records of the species were assigned with grade D (Insufficient Independent Sources).

Panels e and f in Figure 7 show two examples of three nodes subgraphs, where specimens were graded as E. In panel e, the species *Ammotragus lervia* links to two clusters: BOLD:ADC6688 and BOLD:ADQ2389, being the divergence distance between them equal to 5.26%. In panel f, the species *Bos mutus* and *Bos grunniens* are clustered into one single cluster, BOLD:AAD9483.

Figure 7g displays a more complex situation of an isolated subgraph containing 22 nodes: 10 genetic clusters and 12 species (9 graded with E and 3 with D).

The *Bovidae* database showed to be especially hard to audit and curate since it contains many species linked to distant clusters, as well as several clusters containing specimens belonging to two or more species. Furthermore, some of the isolated subgraphs are extremely complex, containing several species nodes connected to different cluster nodes (e.g., Figure 7g). Such complex situations (subgraphs) are not straightforward to fix simply by deleting or editing some records (links).

Problems in this database are deeper and need further review by experts in *Bovidae* taxonomy. Nevertheless, some remarks resulting from the visual inspection of the subgraph presented in Figure 7g should be made. One of the specimens of *Bos taurus* species is grouped in the cluster BOLD:AAE0841 with specimens from different genus such as *Bubalus bubalis*, *Bubalus quarlesi*, or *Bubalus arnee*. Even stranger is the link connecting 7 *Bos taurus* specimens to cluster BOLD:ACB3692, which contains also 18 specimens from a different subfamily, *Pseudois*. The existence of species with specimens clustered in distant clusters is illustrated, e.g., by specimens of *Antilope cervicapra* being gathered in two clusters that diverge by 8.55%. Interestingly, one of the clusters, BOLD:ADC5992, also connects to specimens labeled as *Saiga tatarica*, which belong to different subfamilies.

#### 3.2.2. Felidae Family

The 565 *Felidae* records represent 37 species that group into 54 clusters. Unlike what occurred for the *Bovidae* dataset, the majority of the *Felidae* records was graded with A or B, although several being graded as E—see Table 2.

Editing and/or removing records from a library must result from the application of specific criteria defined by a skilled auditor, since they depend on factors that may vary with the taxa under evaluation. As stated by Costa et al. [13] possible sources of errors may include erroneous morphological taxonomy, poor molecular-based evidence of the species boundaries, inaccuracies during the sequencing pipeline, synonyms or erroneous species names, or mislabeling. All these possible sources of errors should be considered before applying any edition to the dataset.

In a previous work [11], two standardized corrections were suggested that could be implemented. The first was the removal of all records graded as D, as, by definition, there are not enough data in the library to ensure reliability and reproducibility according to the best forensic practices. The second correction was to remove all single source edges that contradict other records (e.g., single source edges that act as bridges connecting subgraphs that otherwise would be separated).

**Figure 8:**
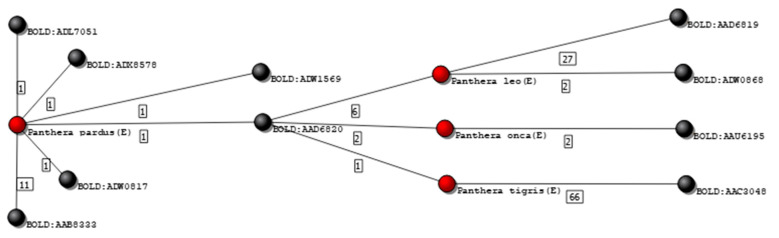
Isolated graph of species *P. pardus*, *P. leo*, *P. onca,* and *P. tigris*. Nodes colored in black represent genetic clusters, and nodes colored red represent species graded as E.

Figure 8 shows an isolated subgraph containing 4 species connected to 10 clusters. One of these species is the *Panthera tigris*, with 67 records from 16 different sources. Among those, one record was grouped in a cluster (BOLD:AAD6820) that did not contain any other *P. tigris* specimen. Inspecting that specific record, it was possible to verify that it was mined from GenBank (accession number KX012651), in which is declared as being part of an unpublished work. Deleting this single record from the database automatically changed the grading of the remaining 66 records of the *P. tigris* for upper grades. Table 2 shows the frequency of records in each grade, before and after editing the *Felidae* database, according to the two previously described criteria.

## 4. Discussion

The case studies presented in this work demonstrate the need and relevance of 4SpecID for auditing and annotating databases. However, some of the examples shown in this work deserve further considerations.

For instance, in *Bovidae* analysis, the cluster BOLD:ACB3692 showed to contain specimens from four species: 7 records from *B. taurus* and 18 from three species of the subfamily *Pseudois*. It is noteworthy that all 7 *B. taurus* specimens were mined from GenBank (accession numbers from JX120616 to JX120623) and belong to one single source, classified as “unpublished.” Considering these facts and the obvious morphological differences between *B. taurus* and the alternative taxa, these sequences do not seem to present the required degree of reliability for a reference library and should be removed or, at least, flagged to avoid future DNA-based misidentifications. However, the removal of these records from the database does not change the grade assigned to the remaining records in Figure 7g. This example clearly illustrates the need of multiple independent sources of information, in order to confer reliability to any given annotation.

Another point that should be specifically mentioned is related to the 111 records graded as D, also in *Bovidae* database. Some of these records could have been assigned to grade A (e.g., *Procapra picticaudata*) if the requirement of at least two sources had been relaxed, by setting the parameter n, number of independent sources of data, to 1 at the beginning of the project. Again, as the previous example clearly demonstrates, in the absence of independent validation, discarding of single source records from the dataset (regardless of the number of records) should be considered or, at least, those records should be flagged to guarantee that any user understands the risk of assuming the information as valid.

The degree of difficulty in auditing different datasets is highly variable, as it was shown when *Bovidae* and *Felidae* are compared. Indeed, the dataset of the *Felidae* family showed to have, at the beginning of the 4SpecID pipeline, large proportions of extreme grades: either A or E. Nevertheless, most of the lowest grades resulted from single source nodes that acted as bridges between otherwise perfectly defined species-cluster pairs. This showed to be also the case for *Canidae* in [11]. In these cases, the removal of few records may have a major impact in the dataset grading. On the contrary, intricate and complex dataset graphs, as occurred for *Bovidae* family, may be due to inaccuracies in the data uploaded but also to the existence of closely related species, including wild and domesticated taxa, sometimes hybridizing [14].

When performance issues and its portability across different computing platforms are analyzed, it is clear that software applications must be professionally developed, with the right development tools. Only this way is possible to make the best use of the available capabilities that current computing systems offer, with a special focus on the number of computing elements (or cores) that currently populate the CPU chips and their capacity to efficiently handle matrix operations. This is not only present on high performance computer systems (HPC) but also in laptops (and moving fast into smartphones).

Moreover, 4SpecID works with any reference library but has specific data mining functions to work with reference libraries from BOLD. Its scalability enables the auditing and annotation of large datasets by taking advantage of enhanced parallel features. The main bottleneck of the grading algorithm is the internet communication to BOLD database when necessary. Nevertheless, this communication is only required for libraries downloaded from BOLD, and for the first time, a given BIN is evaluated. When the clusters divergence distance is saved locally (either after the first automatic download from BOLD or using non-BOLD reference libraries), the grading algorithm run-time decreases by several orders of magnitude.

## 5. Conclusions

4SpecID is a user-friendly, freely available, software tool helping to semiautomatically audit and annotate DNA datasets for taxonomic identification, specifically designed for forensic applications. In accordance to forensic standards considering the reproducibility and validation of data, the first criterion for grading the records of a given taxon was set on the number of independent sources. Specifically, 4SpecID is the only software tool for auditing and annotation of reference DNA libraries that enables the direct edition of unreliable records. Furthermore, the performance results of the tool execution time are also quite remarkable when compared to its direct competition, especially after distance matrices are built and locally saved.

Both the source code and the binary files for laptop/desktop operating systems are available at http://4SpecID.github.io, as well as tutorials and sample files. Providing both versions of the software ensures that users with different needs and degrees of computing expertise can use the tool, for different goals. Moreover, 4SpecID is primarily targeted to noninformatics experts, allowing an easy installation and usability of the binary files. On the other hand, by releasing the source code, the future integration of 4SpecID as a module in bigger pipelines is facilitated, as well as its code modification or extension with new features. This will also allow the validation of the source code, again in compliance with forensic golden standards.

Furthermore, 4SpecID was designed to efficiently annotate and audit any public or private reference DNA library, providing results in an accurate and very fast way. Nevertheless, given its importance in the field, it has specific data mining functions that were tuned to automate the auditing of BOLD systems databases. Besides its primary goal, 4SpecID has potential to be used for other scientific purposes, such as to guide researchers to easily identify morphospecies that may have diverged. By the simple observation of the isolated subgraphs, species connected to more than one distant cluster may be the focus of further investigation to discover the reason behind the genetic divergence. Conversely, if more than one morphospecies are grouped in one single genetic cluster, three possibilities should be investigated: i) errors in the species identification have occurred; ii) a poor choice of parameters in the clustering algorithm that originated divergence distances not compatible to the actual species boundaries, and iii) the existence of an unique species designation for more than one species (cryptic species), which is a possibility of utmost relevance, namely, for conservation, deserving further investigation [15,16,17].

## Figures and Tables

**Table 1:** Summary results of Bovidae dataset downloaded from BOLD on 10 November 2020.

Grade	Count	Frequency (%)
A	149	6.32
B	119	5.05
C	11	0.47
D	111	4.71
E	1967	83.45
Total	2357	100

**Table 2:** Summary results of *Felidae* dataset downloaded from BOLD on 17 November 2020.

Grade	Frequency in Database (%)	Frequency After Auditing (%)
A	49.03	62.03
B	10.62	13.02
C	0.71	0.72
D	1.59	0
E	38.05	24.23

## Data Availability

Publicly available datasets were analyzed in this study. This data can be found here: https://github.com/4SpecID/4SpecID/tree/main/Databases.

## References

[1] Amorim A., Budowle B. (2016). Handbook of Forensic Genetics: Biodiversity and Heredity in Civil and Criminal Investigation.

[2] Ratnasingham S., Hebert P.D. (2007). bold: The Barcode of Life Data System (http://www.barcodinglife.org). Mol. Ecol. Notes.

[3] Benson D.A., Cavanaugh M., Clark K., Karsch-Mizrachi I., Lipman D.J., Ostell J., Sayers E.W. (2013). GenBank. Nucleic Acids Res..

[4] Benson D.A., Cavanaugh M., Clark K., Karsch-Mizrachi I., Lipman D.J., Ostell J., Sayers E.W. (2017). GenBank. Nucleic Acids Res..

[5] Oliveira L.M., Knebelsberger T., Landi M., Soares P., Raupach M.J., Costa F.O. (2016). Assembling and auditing a comprehensive DNA barcode reference library for European marine fishes. J. Fish. Biol..

[6] Weigand H., Beermann A.J., Ciampor F., Costa F.O., Csabai Z., Duarte S., Geiger M.F., Grabowski M., Rimet F., Rulik B. (2019). DNA barcode reference libraries for the monitoring of aquatic biota in Europe: Gap-analysis and recommendations for future work. Sci. Total Environ..

[7] Nilsson R.H., Larsson K.-H., Taylor A.F.S., Bengtsson-Palme J., Jeppesen T.S., Schigel D., Kennedy P., Picard K., Glöckner F.O., Tedersoo L. (2018). The UNITE database for molecular identification of fungi: Handling dark taxa and parallel taxonomic classifications. Nucleic Acids Res..

[8] Chaumeil P., Fischer-Le Saux M., Frigerio J.-M., Grenier E., Rimet F., Streito J.-C., Laval V., Franc A. (2018). R-Syst: A network Providing Curated Molecular Databases and Data Analysis Tools for Taxonomy and Systematics (Prokaryotes and Eucaryotes).

[9] Rimet F., Chaumeil P., Keck F., Kermarrec L., Vasselon V., Kahlert M., Franc A., Bouchez A. (2016). R-Syst::diatom: An open-access and curated barcode database for diatoms and freshwater monitoring. Database.

[10] Fontes J.T., Vieira P.E., Ekrem T., Soares P., Costa F.O. (2020). BAGS: An automated Barcode, Audit & Grade System for DNA barcode reference libraries. Mol. Ecol. Resour..

[11] Conde-Sousa E., Pinto N., Amorim A. (2019). Reference DNA databases for forensic species identification: Auditing algorithms. Forensic Sci. Int. Genet..

[12] Ratnasingham S., Hebert P.D. (2013). A DNA-based registry for all animal species: The barcode index number (BIN) system. PLoS ONE.

[13] Costa F.O., Landi M., Martins R., Costa M.H., Costa M.E., Carneiro M., Alves M.J., Steinke D., Carvalho G.R. (2012). A Ranking System for Reference Libraries of DNA Barcodes: Application to Marine Fish Species from Portugal. PLoS ONE.

[14] Amorim A., Pereira F., Alves C., García O. (2020). Species assignment in forensics and the challenge of hybrids. Forensic Sci. Int. Genet..

[15] Pires W.M.M., Barros M.C., Fraga E.C. (2020). DNA Barcoding unveils cryptic lineages of Hoplias malabaricus from Northeastern Brazil. Braz. J. Biol..

[16] Hosegood J., Humble E., Ogden R., de Bruyn M., Creer S., Stevens G.M.W., Abudaya M., Bassos-Hull K., Bonfil R., Fernando D. (2020). Phylogenomics and species delimitation for effective conservation of manta and devil rays. Mol. Ecol..

[17] Lovrenčić L., Bonassin L., Boštjančić L.L., Podnar M., Jelić M., Klobučar G., Jaklič M., Slavevska-Stamenković V., Hinić J., Maguire I. (2020). New insights into the genetic diversity of the stone crayfish: Taxonomic and conservation implications. BMC Evol. Biol..

